# TSG-6 Inhibits Oxidative Stress and Induces M2 Polarization of Hepatic Macrophages in Mice With Alcoholic Hepatitis *via* Suppression of STAT3 Activation

**DOI:** 10.3389/fphar.2020.00010

**Published:** 2020-02-04

**Authors:** Yue-Meng Wan, Hua-Mei Wu, Yu-Hua Li, Zhi-Yuan Xu, Jin-Hui Yang, Chang Liu, Yue-Feng He, Men-Jie Wang, Xi-Nan Wu, Yuan Zhang

**Affiliations:** ^1^Gastroenterology Department, the 2^nd^ Affiliated Hospital of Kunming Medical University, Kunming, China; ^2^Department of Occupational, Labor and Environmental Health, Public Health Institute of Kunming Medical University, Kunming, China; ^3^The Biomedical Engineering Research Center, Kunming Medical University, Kunming, China

**Keywords:** TSG-6, alcoholic hepatitis, oxidative stress, macrophage polarization, STAT3 activation

## Abstract

Tumor necrosis factor (TNF)-α-stimulated protein 6 (TSG-6) is a secreted protein with diverse tissue protective and anti-inflammatory properties. We aimed to investigate its effects in treating mice with alcoholic hepatitis (AH) and the associated mechanisms. AH was induced in 8–10 week female C57BL/6N mice by chronic-binge ethanol feeding for 10 days. Intraperitoneal (i.p.) injection of recombinant mouse TSG-6 or saline were performed in mice on day 10. Blood samples and hepatic tissues were collected on day 11. Biochemistry, liver histology, flow cytometry, and cytokine measurements were conducted. Compared to the normal control mice, the AH mice had significantly increased liver/body weight ratio, serum alanine aminotransferase (ALT) and aspartate aminotransferases (AST), hepatic total cholesterol (TC), triglyceride (TG), malondialdehyde (MDA), hepatic macrophage infiltration, serum and hepatic interleukin (IL)-6, and tumor necrosis factor (TNF)-α, which were markedly reduced by i.p. injection of rmTSG-6. Compared to the normal control mice, the hepatic glutathione (GSH), accumulation of M2 macrophages, serum, and hepatic IL-10 and TSG-6 were prominently reduced in the AH mice, which were significantly enhanced after i.p. injection of rmTSG-6. Compared to the normal control mice, hepatic activation of signal transducer and activator of transcription 3 (STAT3) was significantly induced, which was markedly suppressed by rmTSG-6 treatment. TSG-6 was effective for the treatment of AH mice, which might be associated with its ability in inhibiting hepatic oxidative stress and inducing hepatic M2 macrophages polarization *via* suppressing STAT3 activation.

## Introduction

Alcoholic liver disease (ALD) is a leading cause of mortality with increasing prevalence worldwide (Liangpunsakul et al., 2016). The spectrum of ALD is broad, ranging from alcoholic fatty liver, alcoholic steatohepatitis, alcoholic hepatitis (AH), fibrosis, cirrhosis, and hepatocellular carcinoma (Teschke, 2019). They may coexist in a given patient, and are often classified into three histological stages: alcoholic fatty liver or simple steatosis, AH, and alcoholic cirrhosis (MacSween and Burt, 1986). At the stage of AH, hepatic inflammation takes place, and the outcome is determined by the severity of liver damage (O'Shea et al., 2010a; O'Shea et al., 2010b). Notably, AH also represents a wide spectrum of hepatic lesions, ranging from mild injury to severe and fatal injury, and often presents acutely in the setting of chronic hepatitis (O'Shea et al., 2010a; O'Shea et al., 2010b). Currently, the recommended therapies for severe AH are corticosteroids or pentoxifylline if the patients had ongoing infections or acute renal failure (O'Shea et al., 2010a; O'Shea et al., 2010b; Mathurin et al., 2012). However, the efficacy of treatment with these two types of drugs is still suboptimal with a 6-month mortality being about 30%–40% (Louvet and Mathurin, 2015). As a result, there is urgent need to explore other effective therapies.

Tumor necrosis factor (TNF)-α-stimulated protein 6 (TSG-6) is a secreted protein with diverse tissue protective and anti-inflammatory properties (Day and Milner, 2019). Recently, numerous studies reported that TSG-6 was able to regulate the inflammatory response and promote repair of tissue injuries (Watanabe et al., 2013; Beltran et al., 2015; Wang et al., 2015). Nonetheless, there is limited data in the literature about its efficacy in AH. Signal transducer and activator of transcription 3 (STAT3) is a transcription factor that can be activated by many molecules including interleukin (IL)-6 and its related cytokines, IL-22 and interferon-α/β/γ, and it plays an important role in liver injury, hepatic steatosis, inflammation and regeneration (Wang et al., 2011). However, it remains unclear whether and how TSG-6 can act on hepatic STAT3 signaling in AH.

Therefore, we performed the present study to investigate the efficacy of TSG-6, and to explore its association with hepatic STAT3 signaling in a murine model of AH.

## Materials and Methods

### Animals

A total of 30 female C57BL/6N mice aged 8–10 weeks were supplied by the Experimental Animal Center of Kunming Medical University weighting 17–21 g. All mice were divided into three groups randomly (n = 10), normal control group (NC group), AH mice without treatment group (AH group), and recombinant mouse (rm) TSG-6 treatment group (TSG-6 group). Mice were kept in common animal house with room temperature (21°C~24°C), humidity (40%~70%) and exposure to 12-hour (h) light and dark cycle. All procedures were performed in accordance with the Guideline of Animal Care and Use Committee of Kunming Medical University (KMU). This study has been approved by KMU Ethics Committees (No. KMMU 2019067).

### Experimental AH Model

The AH modeling was performed by feeding mice with control or 5% (v/v) ethanol-containing Lieber-DeCarli liquid diet (TROPHIC Animal Feed High-Tech Co. Ltd, Jiangsu, China) as previously described (Wu et al., 2016) with slight modification in gavage (three in our experiment vs. one in the previous study). Briefly, modeling process consists of a liquid diet adapting period (5 days), modeling period (10 days) with gavages (2 times), gavaging (one time) and sampling (1 day). The AH mice were fed ethanol (5% v/v) containing liquid diets ad libitum for 10 days plus 3 times of ethanol gavage (31.5% (v/v) ethanol, 400 µl/mouse), whereas the normal control mice were pair-fed control diet and gavaged with isocaloric maltose-dextrin [45% (w/v), 400 µl/mouse]. Gavages were always performed in the morning on the 3^rd^, 6^th^, 11^th^ day of modeling period. Both diets were freshly prepared daily. Nine hours after the third gavage, mice were anesthetized and followed by harvesting blood and liver tissues for the further analysis. Serum was obtained and stored at −80°C. Portions of liver tissue were snap-frozen in liquid nitrogen and then stored at −80°C, while others were fixed in 10% neutral-buffered formalin for histological analysis.

### Biochemical Assays

Serum alanine aminotransferase (ALT) and aspartate aminotransferase (AST) levels, hepatic total cholesterol (TC), triglyceride (TG), malondialdehyde (MDA), and glutathione (GSH) concentrations were measured using spectrophotometric assay kits (NanJing JianCheng Bioengineering Institute, Jiangsu, China) according to the manufacturer's instructions.

### Liver Histology

Liver samples were fixed in 10% paraformaldehyde, embedded in paraffin, and cut into 5 µm sections. The sections were then subjected to dewaxing, hydration and hematoxylin and eosin (H&E) staining, and oil red O staining.

### Flow Cytometric Analysis

The flow cytometric analysis of macrophage status in liver tissues was performed as previously described (Bertola et al., 2013). The total number of macrophages and M2 macrophages that infiltrated the hepatic tissue can be measured using antibodies specific to CD11b and CD206, respectively (Song et al., 2018). The following antibodies were used: rat against mouse CD11b-allophycocyanin (APC; Abcam, Cambridge, MA, USA) and anti-CD206-phycoerythrin (PE; Invitrogen, Raritan, NJ, USA). Flow cytometry was performed on a Partec GmbH CyFlow Space system (Partec, Augsburg, Germany).

### Real-Time Quantitative Polymerase Chain Reaction (RT-qPCR)

RT-qPCR was performed and analyzed as previously described (Li et al., 2018). Total RNA from liver tissues was extracted using TRIzol™ Reagent (Invitrogen, Carlsbad, CA, USA). Total RNA was reverse-transcribed to cDNA using the RevertAidTM First Strand cDNA Synthesis Kit (Thermo Fisher Scientific, Waltham, MA, USA). PCR was performed in triplicate for each sample on the ABI stepone plus real-time PCR System (Applied Biosystems, San Francisco, CA, USA) using the SYBR Green master mix (KAPA, Wilmington, MA, USA). The running procedure was 10 min at 95°C, 40~45 cycles of 15 s at 95°C and 30 s at 60°C, followed by a melt curve. Gene expression was quantified by the standard 2^−ΔΔCt^ method using β-actin as an internal control. The PCR primer sequences were listed in **Table 1**.

**Table 1:** Real-time PCR primers used in this study.

Primer name	Primer sequence	
IL-6	Forward	ACCTGTCTATACCACTTC
	Reverse	GCATCATCGTTGTTCATA
TNF-α	Forward	TTCTGTCTACTGAACTTC
	Reverse	CCATAGAACTGATGAGAG
IL-10	Forward	AGCAGGTGAAGAGTGATT
	Reverse	GCAGTTGATGAAGATGTCA
TSG-6	Forward	CTTGGCTGACTATGTAGA
	Reverse	TTCCTGTGCTAATGATGT
β-actin	Forward	TATGGAATCCTGTGGCATC
	Reverse	GTGTTGGCATAGAGGTCTT

IL, interleukin; TNF, tumor necrosis factor; TSG-6, TNF-α stimulated gene/protein-6.

### Enzyme-Linked Immunosorbent Assay (ELISA)

The serum levels of interleukin (IL)-6, tumor necrosis factor (TNF)-α, IL-10 (Elabscience Biotechnology Co., Ltd, Wuhan, China), and TSG-6 (Shanghai Enzyme-linked Biotechnology Co., Ltd, Shanghai, China) were measured by double antibody sandwich ELISA according to the manufacturers' instructions.

### Western Blot Assay

The liver tissues were split with RIPA lysis bufer (Beyotime, Shanghai, China) and cracked on ice for 20 min. The supernatant was collected after centrifuge at 4, 12,000 rpm/min for 10 min, and the total protein concentration was detected by BCA Kit (Beyotime, Shanghai, China). Proteins (80 μg per lane) were separated by 10% sodium dodecyl sulfate-polyacrylamide gel electrophoresis (SDS-PAGE). After electrophoresis, separated proteins were transferred into polyvinylidene difluoride membrane (PVDF) membrane (Millipore, Billerica, MA, USA) and blocked with 5% skimmed milk powder. The primary antibodies including rabbit anti-mouse IL-6, TNF-α, TSG-6, and STAT3 (Proteintech, Chicago, IL, USA), IL-10 (Beijing Bioss biotechnology co., LTD, Beijing, China), p-STAT3 (Abcam, Cambridge, MA, USA), and β-actin (Zhongshan jinqiao biotechnology co. LTD, Beijing, China) were added and incubated at 4°C overnight after washing with Tris-Buffered Saline and Tween 20 (TBST). On the following day, secondary antibodies, horseradish peroxidase (HRP)-conjugated goat anti-mouse antibodies (Cell Signaling Technology, Beverly, MA, USA) were applied and incubated at room temperature for 1 h after repeated washing with TBST. Enhanced chemiluminescent (ECL) coloration was activated using ECL kit (Millipore, Billerica, MA, USA). Gray values were measured by BIO-RAD Gel Doc XR (Bio-Rad, Hercules, CA, USA). The quantitative assays for relative expression by using Image J software (relative quantity of protein = gray value of targeted protein/gray value of β-actin, triplicated and averaged).

### Statistical Analysis

GraphPad PRISM software, version 8.02 (GraphPad Software Inc, La Jolla, CA, United States) was used for data analysis. Quantitative data were expressed as mean ± standard deviation (SD) in each group. One-way ANOVA followed by Tukey's *post hoc* test was used for mean comparisons in three or more groups. *p* < 0.05 was considered as statistical significance.

## Results

### TSG-6 Treatment Improves Alcohol-Induced Abnormal Liver Function

**Figure 1:**
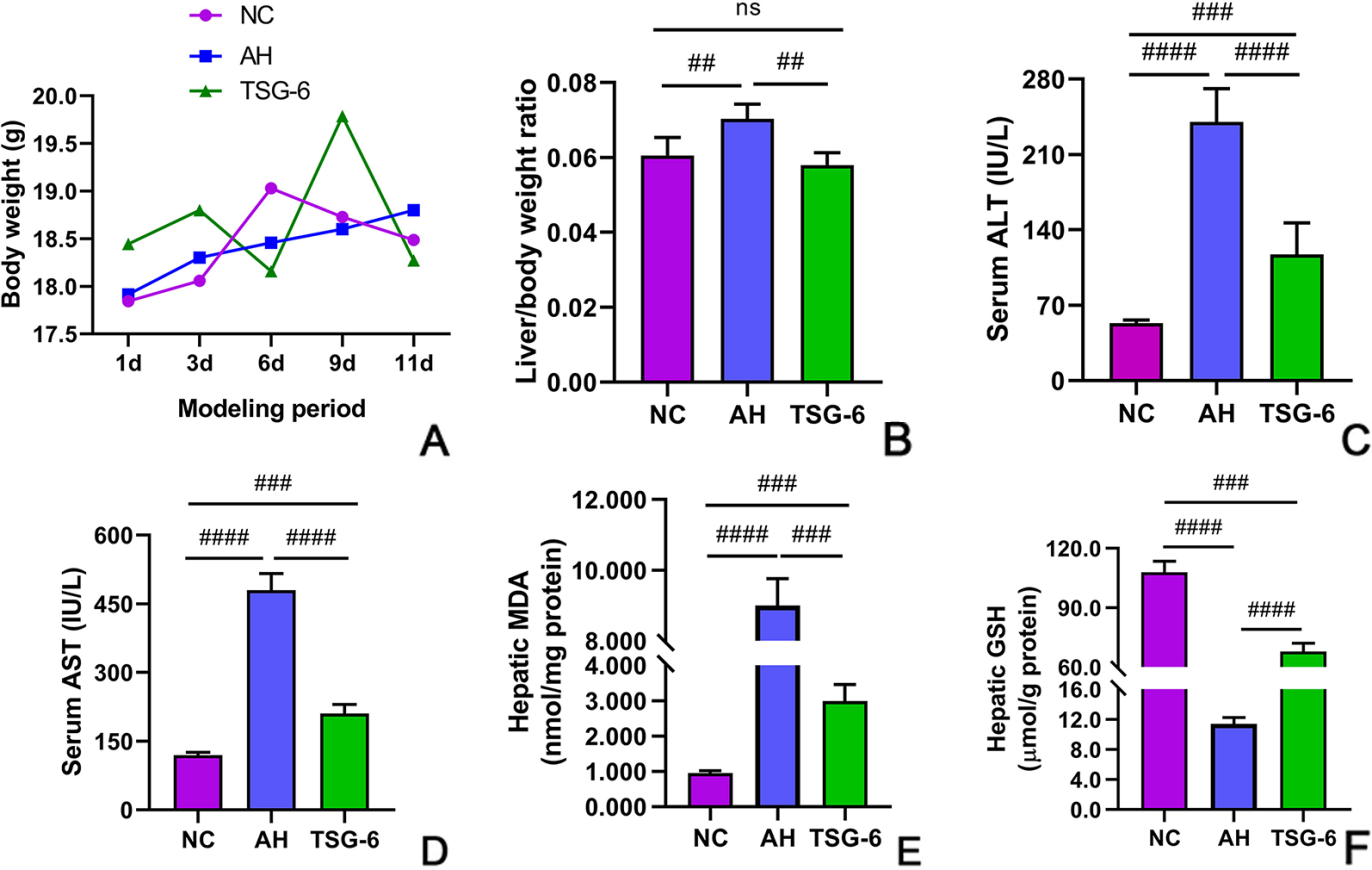
TSG-6 improves alcohol-induced liver injury and alleviates oxidative stress: body weight change **(A)**, liver/body weight ratio **(B)**, ALT **(C)**, AST **(D)**, hepatic MDA **(E)**, and GSH **(F)**. *ns, not significant, ^##^p < 0.01, ^###^p < 0.001, ^####^p < 0.0001*. Six mice were used per group.

At the modeling period, body weights of all mice were measured every three days. The body weights of mice in all groups were similar (**Figure 1A**). As shown in **Figure 1B**, the liver/body weight ratio was highest in the AH group among all groups, but it was comparable between NC group and TSG-6 group at the end of the experiment. Liver injury was evaluated by serum ALT and AST levels. As shown in **Figures 1C, D** both serum ALT and AST levels were significantly increased in mice from the AH group compared to mice from the NC group. However, treatment with rmTSG-6 markedly decreased both serum ALT and AST levels compared to the AH group, though it did not fully normalize them.

### TSG-6 Treatment Alleviates Alcohol-Induced Hepatic Oxidative Stress

Hepatic oxidative stress was evaluated by measuring the hepatic MDA and GSH levels. As indicated in **Figures 1E, F**, mice in the AH group had markedly elevated MDA level but reduced GSH level compared to mice from the NC group. However, treatment with rmTSG-6 significantly reduced the MDA level but enhanced the GSH level when compared to the AH group, although it did not completely rectify them.

### TSG-6 Treatment Attenuates Alcohol-Induced Liver Tissue Damage and Steatosis

**Figure 2:**
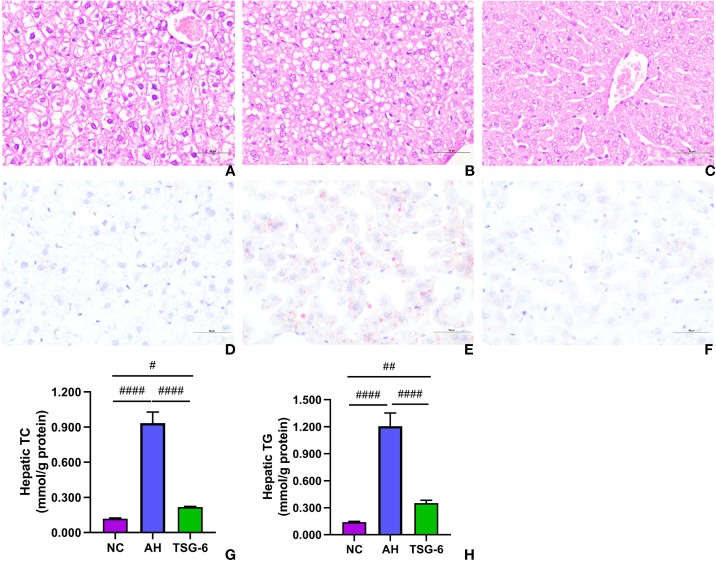
TSG-6 attenuates liver steatosis and hepatic fat accumulation: H&E staining for NC **(A)**, AH **(B)**, TSG-6 **(C)** groups; oil red O staining for NC **(D)**, AH **(E)**, TSG-6 **(F)** groups; hepatic TC **(G)** and TG **(H)** concentrations. Bars = 100 µm, *^#^p < 0.05, ^##^p < 0.01, ^####^p < 0.0001*. Six mice were used per group.

Liver tissue damage and fat accumulation were assessed by liver histology. As presented in **Figures 2A–C**, prominent liver damages in the form of cytoplasmic vacuolation, macro or microvesicular steatosis, loss of cellular boundaries, congestion in the sinusoids, and ballooned hepatocytes were observed in mice from the AH group compared to mice from the NC group. However, treatment with rmTSG-6 markedly alleviated these liver damages but did not completely recover them. As indicated in **Figures 2D–F**, mice in the AH group had obvious fat accumulation (with deeper oil red O staining) compared to mice in the NC group, whereas fat accumulation was markedly reduced after TSG-6 treatment. Hepatic TC and TG measurements further revealed that rmTSG-6 treatment significantly reduced hepatic fat accumulation but did not fully reverse it as noted in liver histology (**Figures 2G, H**).

### TSG-6 Treatment Shifts Hepatic Macrophages Towards a M2 Phenotype

**Figure 3:**
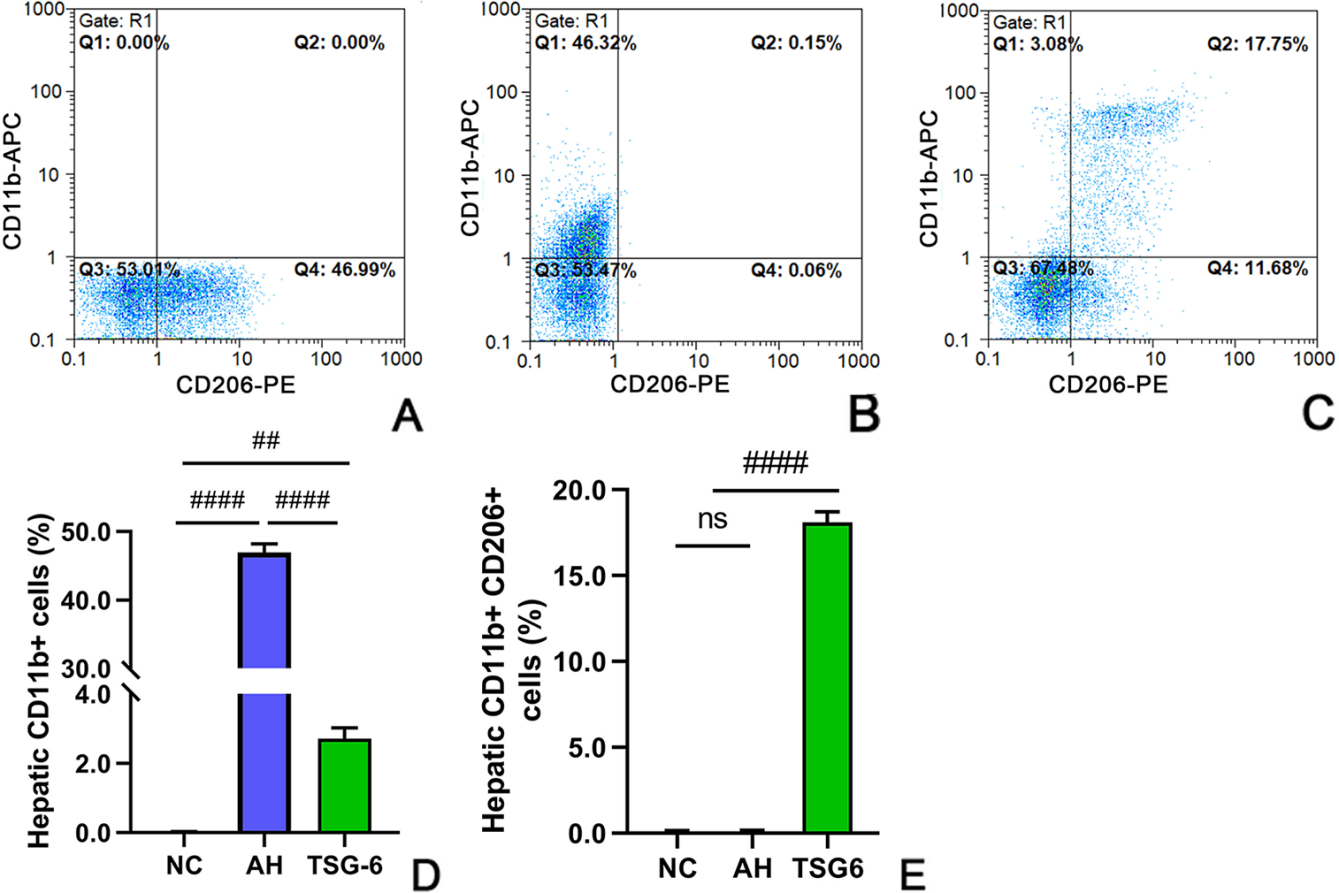
TSG-6 shifts the hepatic macrophages towards a M2 phenotype analyzed by flow cytometry: NC **(A)**, AH **(B)** and TSG-6 **(C)** groups; total hepatic macrophages **(D)**; hepatic M2 macrophages **(E)**. APC, allophycocyanin; PE, phycoerythrin. *ns, not significant, ^##^p < 0.01, ^####^p < 0.0001*. Four mice were used per group.

Hepatic infiltration of macrophages was investigated by flow cytometric analysis. As illustrated in **Figures 3A–E**, there was a significant increase in total macrphages (CD11b+) in mice from the AH group compared to mice from the NC group. However, treatment with rmTSG-6 markedly decreased the total macrphages compared to the AH group but did not entirely reverse them. Conversely, there was a marked decrease in hepatic M2 macrophage (CD11b+CD206+) in mice from the AH group compared to mice from the NC group. However, treatment with rmTSG-6 markedly enhanced the hepatic M2 macrphages but did not completely restore them.

### TSG-6 Treatment Increases Anti-Inflammatory Cytokines But Decreases Proinflammatory Cytokines in Blood

**Figure 4:**
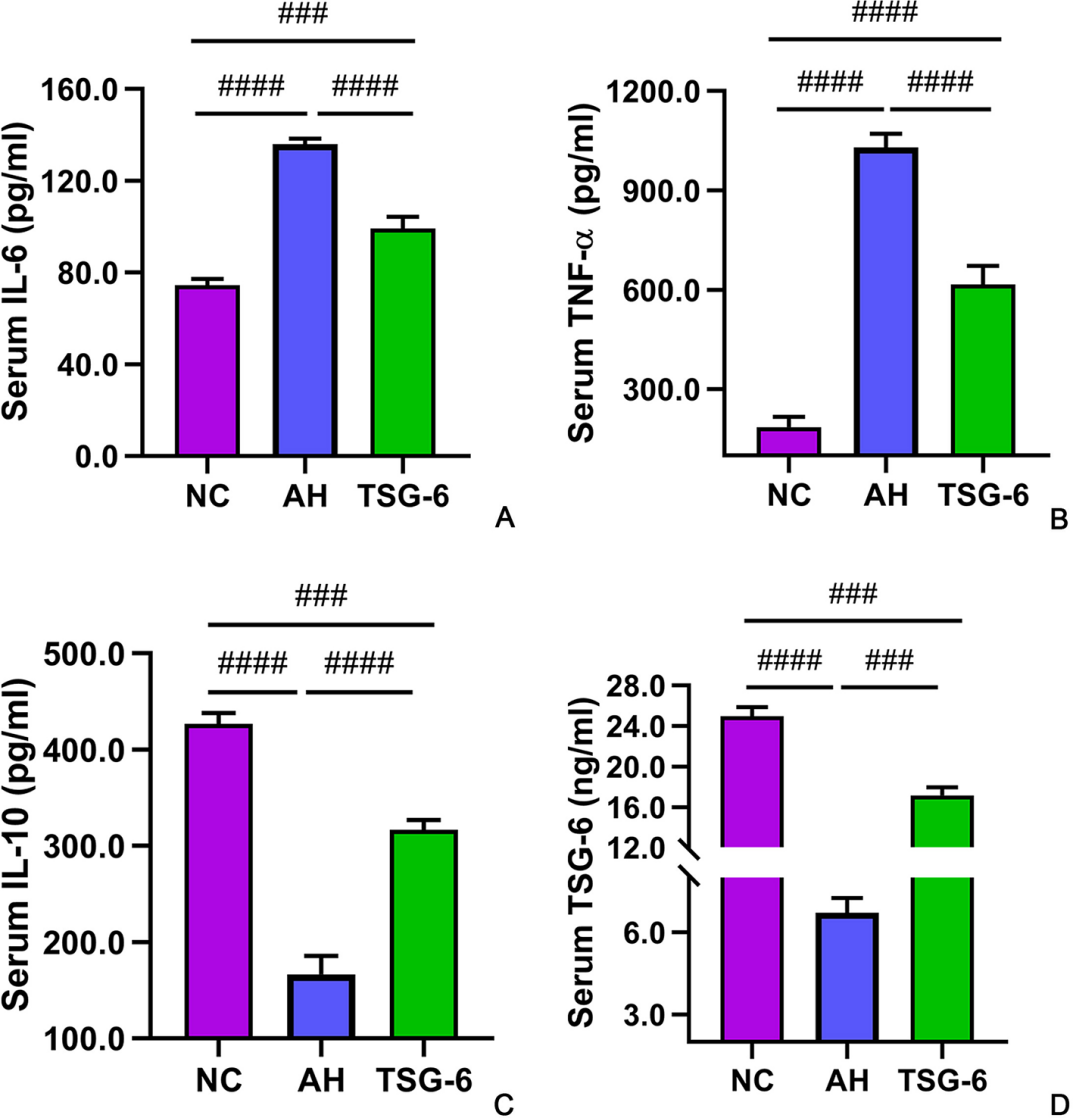
Serum cytokines in all groups of mice. IL-6 **(A)**, TNF-α **(B)**, IL-10 **(C)**, and TSG-6 **(D)**. *^###^p < 0.001, ^####^p < 0.0001*. Five mice were used per group.

The serum cytokines were detected by ELISA tests. As illustrated in **Figures 4A–D**, mice in the AH group had significantly higher IL-6 and TNF-α levels but lower IL-10 and TSG-6 levels compared to mice from the NC group. However, treatment with rmTSG-6 significantly decreased the IL-6 and TNF-α levels but increased the IL-10 and TSG-6 levels compared to the AH group, though it did not fully restore them.

### TSG-6 Treatment Induces Anti-Inflammatory Cytokines But Suppresses Proinflammatory Cytokines in Liver

**Figure 5:**
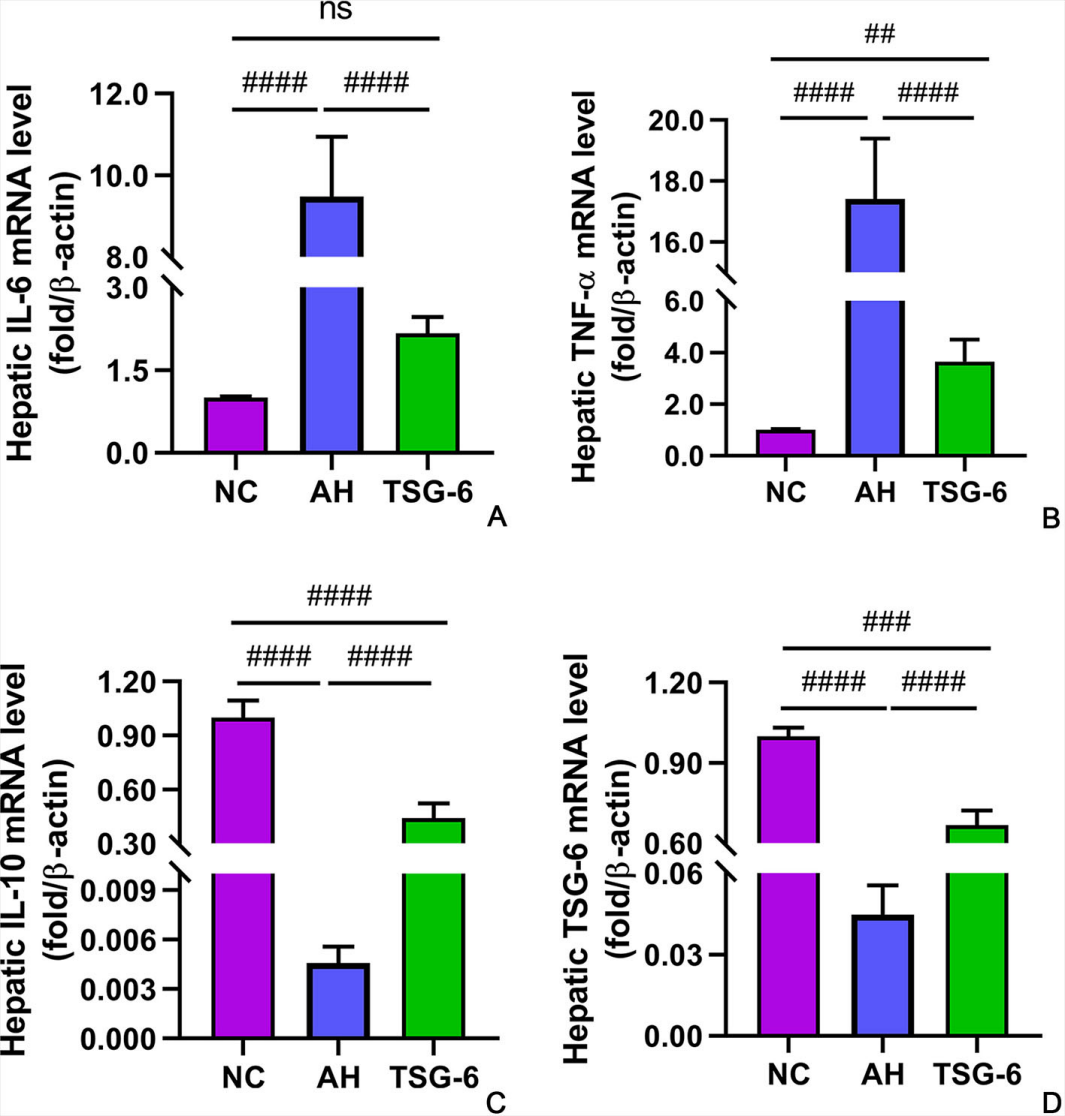
Hepatic expression of cytokines at mRNA levels. IL-6 **(A)**, TNF-α **(B)**, IL-10 **(C)**, and TSG-6 **(D)**. *ns, not significant, ^##^p < 0.01, ^###^p < 0.001, ^####^p < 0.0001*. Five mice were used per group.

**Figure 6:**
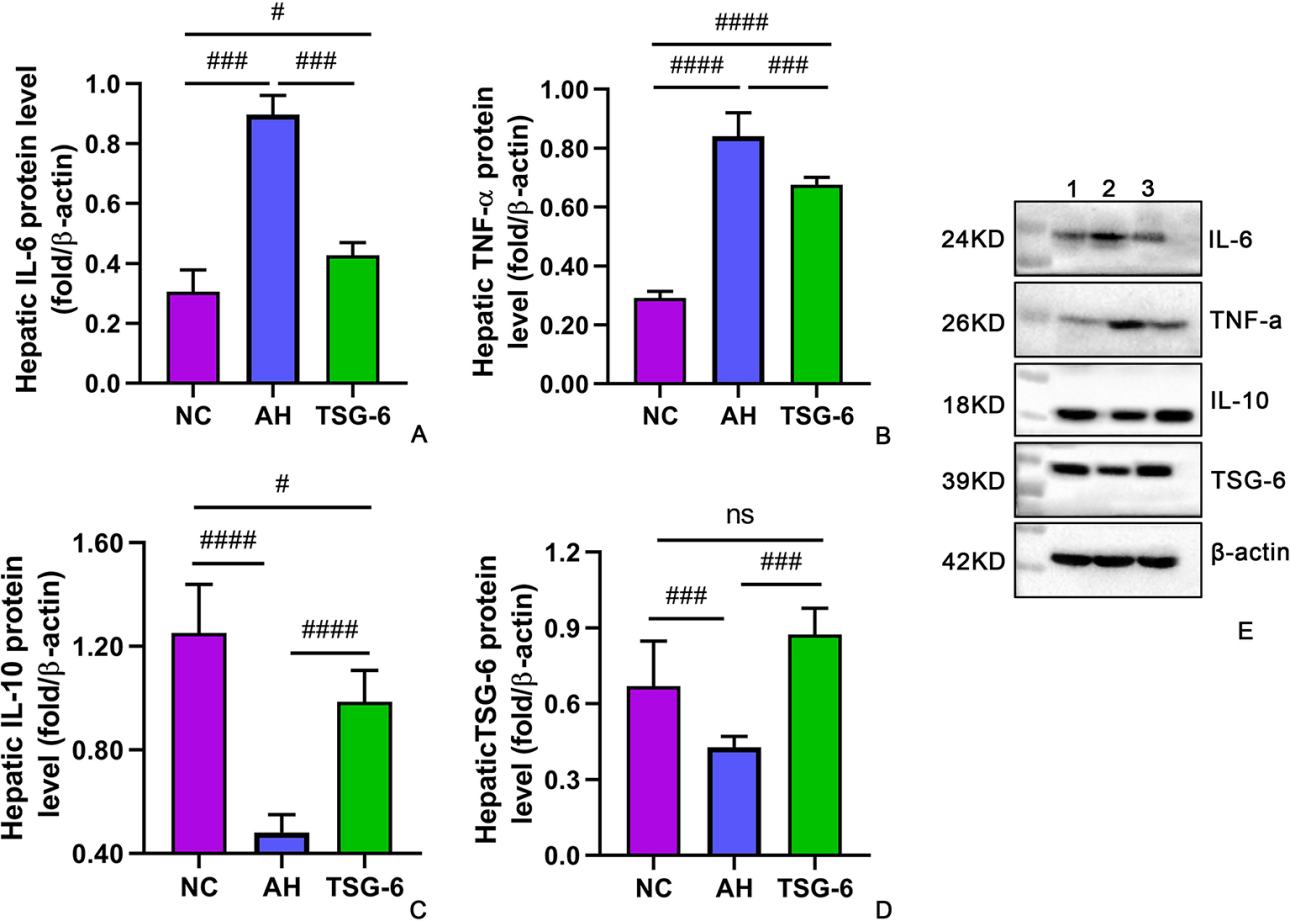
Hepatic expression of cytokines at protein levels. IL-6 **(A)**, TNF-α **(B)**, IL-10 **(C)**, TSG-6 **(D)** and western-blot image **(E)**. *ns, not significant, ^#^P < 0.05, ^###^p < 0.001, ^####^p < 0.0001*. Five mice were used per group.

The hepatic expression of cytokines at mRNA levels and at protein levels were measured by RT-qPCR and Western-blot tests, respectively. As illustrated in **Figures 5A–D**, mice in the AH group had markedly higher IL-6 and TNF-α mRNA levels but lower IL-10 and TSG-6 mRNA levels compared to mice from the NC group. However, treatment with rmTSG-6 significantly reduced the mRNA levels of IL-6 and TNF-α but enhanced the IL-10 and TSG-6 mRNA levels compared to the AH group, though it did not fully reverse all of them excerpt IL-6. As shown in **Figures 6A–E**, similarly, mice in the AH group had marked increment in IL-6 and TNF-α protein levels but reduction in IL-10 and TSG-6 protein levels when compared to mice in the NC group. After treatment with rmTSG-6, the protein levels IL-6 and TNF-α were significantly lowered, but the IL-10 and TSG-6 protein levels were significantly increased compared to the AH group, though they were not completely normalized except TSG-6.

### TSG-6 Treatment Inhibited Hepatic STAT3 Activation

**Figure 7:**
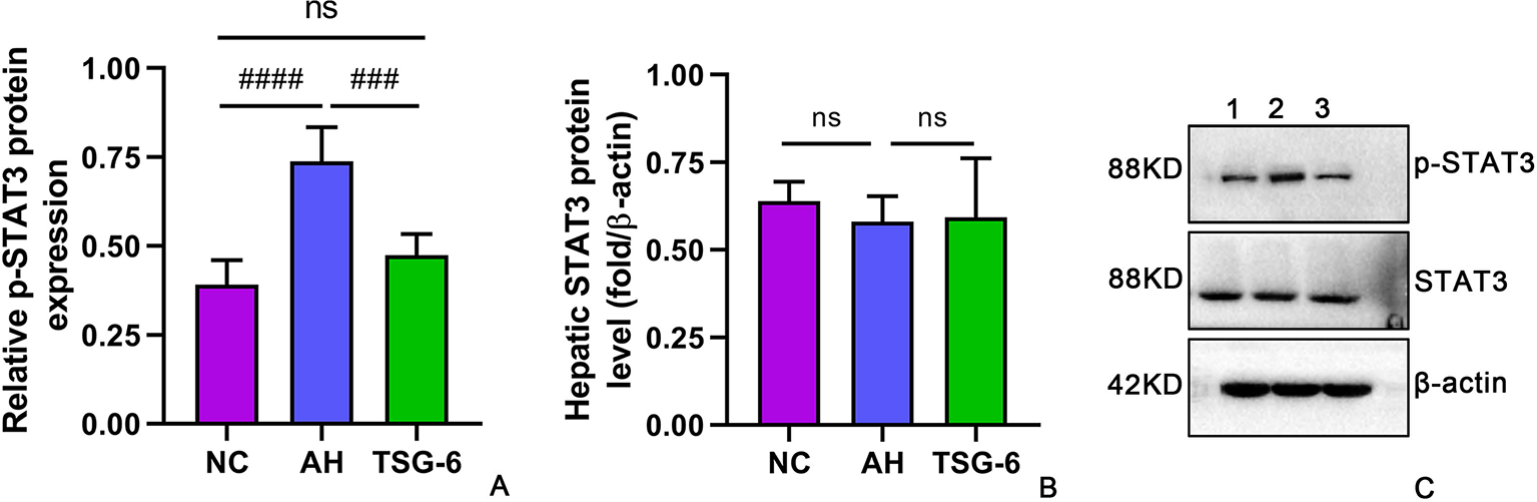
TSG-6 treatment inhibits hepatic levels of phosphorylated STAT3 **(A)** but not STAT3 **(B)**; Western-blot image **(C)**. *ns, not significant, ^###^p < 0.001, ^####^p < 0.0001*. Five mice were used per group.

The mechanism of alcohol-induced liver injury was further investigated by detecting the expression and phosphorylation of the inflammatory signaling molecule STAT3 in mouse livers. As shown in **Figures 7A–C**, there was a marked elevation in phosphorylated form of STAT3 in the AH group compared with the NC group. However, treatment with rmTSG-6 significantly reduced the levels of phosphorylated STAT3 compared to the AH group. Notably, STAT3 levels were not significantly different in all groups.

## Discussion

In this study, three important findings are presented here. First, we found that TSG-6 was effective in treating mice with AH, alleviating the hepatic injury, steatosis, and oxidative stress. Second, we showed that TSG-6 was able to inhibit the total number but induce the M2 type of macrophages infiltrating the livers of AH mice. Third, we demonstrated that TSG-6 could suppress the activation of hepatic STAT3 signaling, which may be the underlying mechanism for this treatment.

The Lieber-DeCarli model of alcoholic liver injury is a widely accepted model to investigate mild or moderate AH (Ki et al., 2010; Bertola et al., 2013; Wu et al., 2016). In line with these studies (Ki et al., 2010; Bertola et al., 2013; Wu et al., 2016), the results of liver/body weight ratio, serum ALT and AST levels, hepatic TG, TC, GSH, and MDA concentrations, histological analyses by H&E and oil red O staining showed that the AH model was successfully established in the present study.

Serum ALT and AST levels are sensitive markers of liver injury. In the present study, we showed that rmTSG-6 treatment caused significant reduction in these two parameters. Hepatic steatosis is a prominent feature in ALD, and it is present in the majority of individuals with ALD (Stickel et al., 2017). In the current study, we demonstrated that hepatic TC and TG contents were significantly reduced after treatment with rmTSG-6, and liver histology using H&E and oil red O staining further supported the alleviation of hepatic steatosis by TSG-6 treatment. Oxidative stress plays a critical role in the pathogenesis of ALD, as numerous studies reported that formation of reactive oxygen species (ROS) are crucial for the progression of ALD (Leung and Nieto, 2013; Bankoglu et al., 2016), because they could induce hepatic steatosis, inflammatory cell infiltration, hepatomegaly, fibrosis and cirrhosis (Li et al., 2014). In this study, we found that treatment with rmTSG-6 caused significant increment in hepatic GSH level but reduction in hepatic MDA, both of which were markers of oxidative stress. Moreover, we also noted that TSG-6 treatment reduced the hepatomegaly in AH mice, as reflected by liver/body ratio. All these results supported that TSG-6 could improve oxidative stress, and alleviate liver injury and hepatic steatosis.

We next examined the hepatic infiltration of macrophages, since activation of peripheral and infiltrating bone-marrow monocytes and macrophages plays a very important role in ALD (McClain et al., 2002; Magdaleno et al., 2017). There are two distinct types of macrophages, M1 and M2 macrophages. M1 macrophages favor inflammatory response, tissue damage and disease progression by producing pro-inflammatory cytokines such as IL-1β, IL-6, IL-12, IL-23, and TNF-α, and by recruiting other proinflammatory cells (e.g., neutrophils) and generating ROS, whereas M2 macrophages favor anti-inflammatory response, tissue repair, and disease resolution by secreting anti-inflammatory cytokines such as IL-10 and transforming growth factor (TGF)-β (Murray and Wynn, 2011; Shapouri-Moghaddam et al., 2018). A recent study reported that a mixed M1 and M2 types of macrophages existed in the blood and liver tissues of mice with AH (Wu et al., 2016). Our data showed that after treatment with rmTSG-6, the total number of macrophages (CD11b+) were markedly inhibited, whereas the number of M2 macrophages (CD11b+CD206+) were prominently induced (**Figure 3**) in livers of AH mice. These data supported that TSG-6 treatment could induce polarization of macrophages towards a M2 phenotype, favoring remission of liver injury and inflammation, which was consistent with previous studies (Mittal et al., 2016; Song et al., 2018). To further substantiate these results, we assessed the cytokines in the blood and liver. In concert with previous studies (Mittal et al., 2016; Wu et al., 2016; Song et al., 2018), our study showed that the serum and hepatic proinflammatory cytokines including IL-6 and TNF-α were significantly diminished. Conversely, the serum and hepatic anti-inflammatory cytokines including IL-10 and TSG-6 were prominently enhanced. All these data provided evidence that TSG-6 induced polarization of hepatic macrophages towards a M2 phenotype and attenuated hepatic inflammation in AH mice.

Currently, little is known about the mechanism underlying the polarization of hepatic macrophages towards a M2 phenotype by TSG-6 in AH mice. Wu et al. (2016) reported that telomerase reverse transcriptase switched macrophages towards M1 phenotype by enhancing nuclear factor (NF)-κB signaling pathway in mice with AH. Since STAT3 activation has been well documented in human and murine model of ALD (Larrea et al., 2006; Horiguchi et al., 2007; Horiguchi et al., 2008), we sought to investigate the relationship between STAT3 activation and rmTSG-6 treatment. Our results showed that AH mice had markedly increased STAT3 activation compared to NC mice (**Figure 7**), which was consistent with previous studies (Larrea et al., 2006; Horiguchi et al., 2007; Horiguchi et al., 2008). Previous studies have reported that TSG-6 exerts its functions by inducing microbial polarization towards M2 phenotype in inflammation-induced brain injury *via* regulating the SOCS3/STAT3 axis, and by shifting bone marrow-derived and pulmonary macrophages from M1 to M2 phenotype after suppression of NF-κB signaling and STAT1 and STAT3 activation (Mittal et al., 2016; Li et al., 2018). In keeping with these studies (Mittal et al., 2016; Li et al., 2018), our study also showed that the hepatic STAT3 activation in mice with AH was markedly dampened following treatment with rmTSG-6 (**Figure 7**). These findings suggest that TSG-6 may also suppress STAT3 activation in AH model to induce polarization of hepatic macrophages towards M2 phenotype.

The above ﬁndings lead to the question of in what cells is STAT3 activated by alcohol? Previously, Horiguchi et al. (Horiguchi et al., 2008) reported that STAT3 activation in hepatocytes functioned as an proinflammatory signal, and knocking out STAT3 in hepatocytes resulted in greater hepatic steatosis, higher serum and hepatic TG levels, but less hepatic infiltration by neutrophils and macrophages, and lower hepatic expression of proinflammatory cytokines (TNF-α, IL-6); In contrast, STAT3 activation in macrophages/neutrophils acted as an anti-inflammatory signal, and knocking out STAT3 in macrophages/neutrophils caused higher serum ALT and AST levels, greater hepatic infiltration by neutrophils and macrophages, and enhanced hepatic expression of proinflammatory cytokines (TNF-α, IL-6); Kupffer cells isolated from ethanol-fed mice with STAT3 knockout in hepatocytes or in macrophages/neutrophils (with reduced STAT3 activation) produced similar or higher amounts of ROS and TNF-α than Kupffer cells from wild-type mice. In addition, STAT3 activation in endothelial cells has been shown to exhibit an anti-inflammatory effect (Kano et al., 2003). In the present study, we demonstrated that STAT3 activation was an proinflammatory signal, because the AH mice displayed greater infiltration by neutrophils and macrophages, and higher hepatic expression of proinflammatory cytokines (TNF-α, IL-6) than the NC mice, and suppression of STAT3 activation by rmTSG-6 led to decreased infiltration by neutrophils and macrophages, and reduced hepatic expression of proinflammatory cytokines (TNF-α, IL-6). These data suggest that, in our study, STAT3 activation is most likely localized in hepatocytes, rather than neutrophils, macrophages, kupffer cells, or endothelial cells; or STAT3 activation occurs in all these cells but is dominant in hepatocytes with the net effect of STAT3 activation being proinflammatory. Notably, according to Horiguchi et al. (Horiguchi et al., 2008), if STAT3 activation is localized within hepatocytes, suppression of STAT3 activation should have resulted in greater hepatic steatosis, and higher serum and hepatic TG levels. In our study, suppression of STAT3 activation by TSG-6 resulted in reduced hepatic steatosis and hepatic TG level, which was at odds with our assumption that STAT3 activation was localized within hepatocytes. This discrepancy may be explained as follows: first, hepatic STAT3 signaling was impaired in ethanol-induced steatosis, and impaired STAT3 signaling could prompt the development of hepatic steatosis (Gao, 2004); second, while our model of AH is induced by chronic-binge ethanol feeding, the model of ALD in Horiguchi et al. is induced by chronic ethanol feeding, and these two models of alcohol-induced liver injury have different liver damage profiles (Bertola et al., 2013). Overall, our findings suggest that ethanol-induced STAT3 activation in the current model of AH is most likely localized in hepatocytes. However, it remains to be clarified how STAT3 activation in hepatocytes induces phenotypic switch of macrophages from M1 to M2.

There are several limitations to the present study. First, we did not analyze the phenotype change of macrophages in mouse peripheral blood, though the serum cytokines supported the polarization of M1 towards M2 type. Second, our mechanistic investigation was not in-depth, and we did not provide direct evidences linking TSG-6, STAT3 activation, and macrophage phenotype polarization, though previous studies had provided robust evidences that TSG-6 could induce polarization of macrophage towards a M2 phenotype in other inflammatory models (Mittal et al., 2016; Li et al., 2018). Third, we did not identify directly the cell types that bore changes in STAT3 activation, and we did not provide sufficient evidence directly linking inhibition of hepatic STAT3 activation by TSG-6 to amelioration of AH in mice. Hopefully, these issues can only be solved in the future studies.

**Figure 8:**
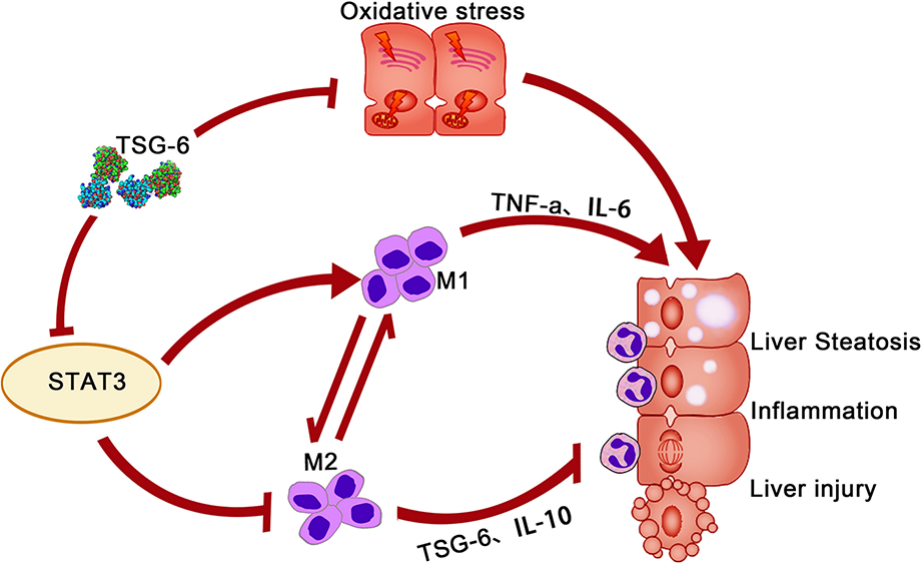
Schematic mechanism of TSG-6 for treatment of alcoholic hepatitis.

In summary, our study clearly demonstrated that TSG-6 was able to improve liver injury, alleviate oxidative stress, attenuate hepatic steatosis, induce polarization of hepatic macrophages towards a M2 phenotype and remission of hepatic inflammation, and suppress hepatic STAT3 activation. These mechanisms may be overlapping and acting together to diminish the liver injury and inflammation in mice with AH (McClain et al., 2002). Based on our results and previous findings (McClain et al., 2002; Gao, 2004; Horiguchi et al., 2008; Bertola et al., 2013; Li et al., 2014; Mittal et al., 2016; Wu et al., 2016; Li et al., 2018; Song et al., 2018), we come up with the schematic mechanism underlying the efficacy of TSG-6 in AH (**Figure 8**), which may provide useful data for clinical researches.

## Data Availability Statement

The datasets used and/or analyzed during the current study are available from the corresponding author on reasonable request.

## Ethics Statement

All animal experiments were approved by the ethics committees of Kunming Medical University (No. KMMU 2019067), and were conducted according to the guidelines of the Animal Care and Use Committee of Kunming Medical University.

## Author Contributions

Y-MW designed the study, performed the experiments, analyzed the data, and wrote the manuscript. CL, Y-FH, M-JW, YZ: carried out the animal experiment, MSCs isolation and culture, RT-qPCR, ELISA and WB assays; collected and analyzed the data; performed the statistical analysis. H-MW, Y-HL, Z-YX, J-HY: carried out biochemical tests, MPO activity analysis, liver histology and immunohistochemistry assays; collected and analyzed the data. X-NW: designed and supervised the study; performed the statistical analysis; edited the manuscript. All authors read and approved the final manuscript.

## Funding

This work was supported by the National Natural Science Foundation of China (NSFC) [Grant Number 81560525 and 40115048].

## Conflict of Interest

The authors declare that the research was conducted in the absence of any commercial or financial relationships that could be construed as a potential conflict of interest.
