# Corrigendum: TSG-6 Inhibits Oxidative Stress and Induces M2 Polarization of Hepatic Macrophages in Mice With Alcoholic Hepatitis *via* Suppression of STAT3 Activation

**DOI:** 10.3389/fphar.2020.00569

**Published:** 2020-05-05

**Authors:** Yue-Meng Wan, Hua-Mei Wu, Yu-Hua Li, Zhi-Yuan Xu, Jin-Hui Yang, Chang Liu, Yue-Feng He, Men-Jie Wang, Xi-Nan Wu, Yuan Zhang

**Affiliations:** ^1^Gastroenterology Department, The 2nd Affiliated Hospital of Kunming Medical University, Kunming, China; ^2^Department of Occupational, Labor and Environmental Health, Public Health Institute of Kunming Medical University, Kunming, China; ^3^The Biomedical Engineering Research Center, Kunming Medical University, Kunming, China

**Keywords:** TSG-6, alcoholic hepatitis, oxidative stress, macrophage polarization, STAT3 activation

In the original article, there was a mistake in **Figure 2** as published. Two microphotos (A, C) were mistakenly inserted. The corrected **Figure 2** appears below.

**Figure 2:**
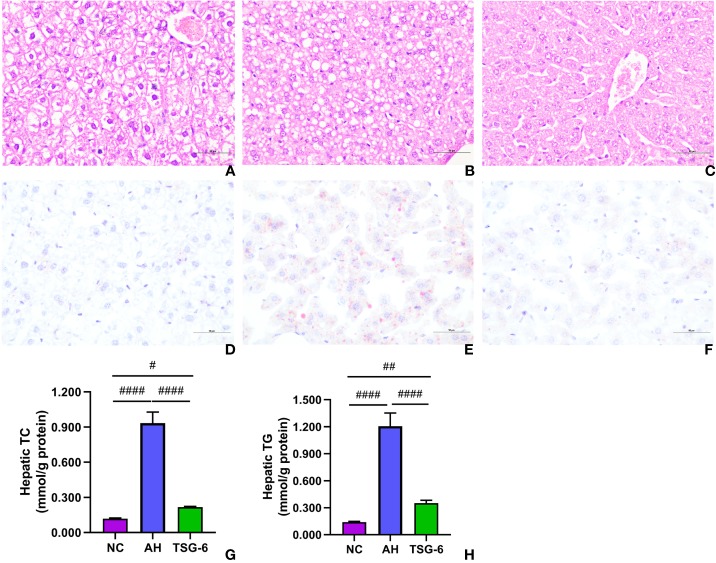
TSG-6 attenuates liver steatosis and hepatic fat accumulation: H&E staining for NC **(A)**, AH **(B)**, TSG-6 **(C)** groups; oil red O staining for NC **(D)**, AH **(E)**, TSG-6 **(F)** groups; hepatic TC **(G)** and TG **(H)** concentrations. Bars = 100 µm, ^#^*p* < 0.05, ^##^*p* < 0.01, ^####^*p* < 0.0001. Six mice were used per group.

In addition, there were two errors in the Abstract. In the second sentence, the word “effective” was used. The correct word is “effects.” In the tenth sentence, the word "were" was used. The correct word was "was." Corrections have been made to the Abstract:

“Tumor necrosis factor (TNF)-α-stimulated protein 6 (TSG-6) is a secreted protein with diverse tissue protective and anti-inflammatory properties. We aimed to investigate its effects in treating mice with alcoholic hepatitis (AH) and the associated mechanisms. AH was induced in 8–10 week female C57BL/6N mice by chronic-binge ethanol feeding for 10 days. Intraperitoneal (i.p.) injection of recombinant mouse TSG-6 or saline were performed in mice on day 10. Blood samples and hepatic tissues were collected on day 11. Biochemistry, liver histology, ﬂow cytometry, and cytokine measurements were conducted. Compared to the normal control mice, the AH mice had significantly increased liver/body weight ratio, serum alanine aminotransferase (ALT) and aspartate aminotransferases (AST), hepatic total cholesterol (TC), triglyceride (TG), malondialdehyde (MDA), hepatic macrophage infiltration, serum and hepatic interleukin (IL)-6, and tumor necrosis factor (TNF)-α, which were markedly reduced by i.p. injection of rmTSG-6. Compared to the normal control mice, the hepatic glutathione (GSH), accumulation of M2 macrophages, serum, and hepatic IL-10 and TSG-6 were prominently reduced in the AH mice, which were significantly enhanced after i.p. injection of rmTSG-6. Compared to the normal control mice, hepatic activation of signal transducer and activator of transcription 3 (STAT3) as significantly induced, which was markedly suppressed by rmTSG-6 treatment. TSG-6 was effective for the treatment of AH mice, which might be associated with its ability in inhibiting hepatic oxidative stress and inducing hepatic M2 macrophages polarization *via* suppressing STAT3 activation.”

The authors apologize for these errors and state that they do not change the scientific conclusions of the article in any way. The original article has been updated.

